# Water as a Prophylactic and a Remedy

**Published:** 1880-10-20

**Authors:** S. G. Webber


### WATER AS A PROPHYLACTIC AND A REMEDY.

BY S. G. WEBBER, M. D.

The subject of water-drinking, he thought, seemed worthy of more than a passing notice. A moderate quantity of fluid taken at meals he considered rather beneficial, while the abstinence advocated by many was injurious. In patients often classed as hypochondriacal or hysterical, where there was no well-defined disease, but only a sense of unrest and discomfort—sometimes amounting to pain—in various locations, and ordinarily accompanied by constipation, it had long been his custom to inquire about the amount of drink they took and the quantity of urine passed by them. Often the former was much below the average, and there was a tendency to dryness of the skin, while the urine was scanty, high-colored and strongly acid, and sometimes deposited a sediment on standing. Under the use of an increased amount of water the perspiration was increased, the urine became more natural and the unpleasant symptoms diminished or altogether disappeared.

During comparatively good health the amount of blood in the system was maintained at nearly the same figure, only so much water being lost through the skin, lungs and kidneys as could be restored from other sources. If too little water were ingested the balance each day against health was very slight, but finally there would be such an accumulation of used-up material that nutrition would be interfered with and unpleasant symptoms developed. If the person continued to eat heartily the surplus part would either pass off by the intestines or be deposited in the shape of fat, the nitrogenized portions of it assisting to load the urine with urea and urates. If such an individual were to drink more water, a larger amount of waste products would be taken up to be eliminated, and so there would be more disintegration of the tissues, while nutrition would be increased.

After describing the favorable effect of water upon the processes of digestion, the writer went on to inquire how much of it an adult should drink in the twenty-four hours. The quantity of liquid required as drink, he believed, would vary slightly with the activity of the skin and the character of the food taken. The amount of drink necessary, as stated by Dalton, was about 52 ounces, or 3.38 pints, while patients repeatedly told him that they drank only a pint or a pint and a half of fluid in a day. When an individual had for months and years averaged an insufficient amount of drink in the twenty-four hours, and the system had become charged with used-up material, it would not perhaps be wise immediately to administer large draughts, whether of ordinary water or of the mineral waters, but the quantity could be rapidly increased until the normal average had been exceeded, which for a while would be attended with advantage. Dr. Webber then related a case treated at the Boston City Hospital, which afforded an interesting example. The patient was a man sixty years of age, who said he had had rheumatism at times since he was a boy, and rheumatic fever seven years before. For more than ten years he had noticed a red sandy sediment in the vessel after micturition, which was frequent, while the quantity of urine passed was scanty. He was a large, fleshy man, with a very large tympanitic abdomen, and said that he suffered from severe pains in the lower part of the back and the hips, numbness in the left leg, and considerable shortness of breath in going up-stairs. He had an idea that he had disease of the heart and kidneys, with dropsy, but there was nothing of the kind present, and no attempt was made to record all his complaints. On September 3d, 4th, 5th and 6th, he passed twenty, twenty-eight, twenty-nine and eighteen ounces of urine respectively. He was told to drink water more freely, and was treated with fluid extract of buchu, when the amount of urine increased to forty, fifty, sixty and sixty-eight ounces on four consecutive days, while his discomfort became greatly diminished, and he expressed himself as feeling much relieved. The writer also mentioned the case of a lady, the subject of very distressing nervous symptoms, who had restricted herself to a cup of tea night and morning, which was the only fluid that she took, and of a physician who was suffering from many symptoms referable to over-work. He found that the latter drank very little also, and was troubled with constipation. In his case the diminished supply of fluid was not the only cause of difficulty, but, in addition to other measures, he was advised to drink more. When seen again, after eight months, he stated that the increase in the amount of fluid ingested had been beneficial, and that he was less constipated.

Human nature is such that if the doctor told his patient to drink two or three pints of Cochituate or Croton water a day, in addition to his tea or coffee, he would be likely to rebel; but if he were instructed to take that amount of Poland or Allandale, or some other similar water, he would forthwith have his keg of mineral water on tap, and drink it in the firm faith that in some mysterious way it would relieve him.

In conclusion, the writer stated his belief that the insufficient ingestion of water was often a predisposing or even exciting cause of many diseases. He had found that a very large proportion of those who suffered from nervous exhaustion did not drink enough. He believed that it was an American peculiarity to ingest too little fluid, and thought that this fact might partly explain the prevalence of neurasthenia in this country. He considered also that one reason of the success of the treatment adopted by Dr. Weir Mitchell, and advocated by him in his "Fat and Blood, and How to Make Them," was to be found in the large amount of milk which he gave his patients. It was not to be expected, however, that in all cases the mere increase of fluid ingested would cure. Too frequently the tissues had been so long illy nourished that that simple plan was not sufficient, so that the time to work the greatest cures with water was before the disease had begun.—*Transactions American Neurological Association, Boston.*

---

**A Good Move.**—The Legislature of South Carolina has passed a law allowing physicians $10 for testifying as experts in any medical case. This is an addition to the per diem and mileage accorded to ordinary witnesses. The law was passed through the effort and agitation of the Abbeville Medical Society.—*Mich. Med. News.*

